# Fat-tail allele-specific expression genes may affect fat deposition in tail of sheep

**DOI:** 10.1371/journal.pone.0316046

**Published:** 2024-12-27

**Authors:** Hossein Mansourizadeh, Mohammad Reza Bakhtiarizadeh, Luciana Correia de Almeida Regitano, Jennifer Jessica Bruscadin

**Affiliations:** 1 Department of Animal and Poultry Science, College of Aburaihan, University of Tehran, Tehran, Iran; 2 Embrapa Southeast Livestock, São Carlos, Brazil; 3 Department of Genetics and Evolution, Federal University of São Carlos (UFSCar), São Carlos, Brazil; South China Agricultural University, CHINA

## Abstract

Different sheep breeds show distinct phenotypic plasticity in fat deposition in the tails. The genetic background underlying fat deposition in the tail of sheep is complex, multifactorial, and may involve allele-specific expression (ASE) mechanism to modulate allelic expression. ASE is a common phenomenon in mammals and refers to allelic imbalanced expression modified by cis-regulatory genetic variants that can be observed at heterozygous loci. Therefore, regulatory processes behind the fat-tail formation in sheep may be to some extent explained by cis- regulatory variants, through ASE mechanism, which was investigated in the present study. An RNA-Seq-based variant calling was applied to perform genome-wide survey of ASE genes using 45 samples from seven independent studies comparing the transcriptome of fat-tail tissue between fat- and thin-tailed sheep breeds. Using a rigorous computational pipeline, 115 differential ASE genes were identified, which were narrowed down to four genes (*LPL*, *SOD3*, *TCP1* and *LRPAP1*) for being detected in at least two studies. Functional analysis revealed that the ASE genes were mainly involved in fat metabolism. Of these, *LPL* was of greater importance, as 1) observed in five studies, 2) reported as ASE gene in the previous studies and 3) with a known role in fat deposition. Our findings implied that complex physiological traits, like fat-tail formation, can be better explained by considering various genetic mechanisms, which can be more finely mapped through ASE analyses. The insights gained in this study indicate that biallelic expression may not be a common mechanism in sheep fat-tail development. Hence, allelic imbalance of the fat deposition-related genes can be considered a novel layer of information for future research on genetic improvement and increased efficiency in sheep breeding programs.

## Introduction

Sheep were likely the first livestock species to be domesticated in the Fertile Crescent about 11,000 years ago [1]. Throughout a long history of evolution and breeding and based on their tail phenotype, sheep have been classified into one of the following main categories: thin- and fat-tailed breeds. The most probable wild ancestors of domestic sheep were thin-tailed. Hence, it is believed that the fat tail phenotype evolved after domestication as a valuable energy reservoir as well as an adaptive response to harsh environmental conditions [2]. However, fat-tail importance is decreasing in modern- or semi-intensive sheep production systems since it decreases the feed efficiency. Furthermore, most consumers prefer lean meat as a healthier choice [3, 4]. In this context, elucidating the genetic mechanisms underlying fat deposition in tail of sheep is of great importance to improve fat-tailed breeds.

The current transcriptome studies based on RNA-Seq datasets measure gene expression by counting the number of mRNA copies of the genes and compare the expression patterns between two or more conditions to identify differentially expressed genes (DEGs). While in diploid mammalian cells, most of the autosomal genes are typically expressed similarly from both alleles, cis-regulatory genetic variants or epigenetic modifications may induce imbalanced expression of the alleles, resulting in allele-specific expression (ASE) [5]. Therefore, ASE, or allelic imbalance, refers to the imbalanced expression of the alleles of a gene in a heterozygous locus, that can increase the diversity of transcript and protein isoforms abundance from one gene [6]. The extreme form of ASE is genomic imprinting or parent-of-origin specific gene expression, which results in the systematic monoallelic expression of the copy inherited from only one parent [7]. It is well known that a significant proportion of phenotypic diversity in mammals is mediated by transcriptional regulation [8, 9] of genes, which directly or indirectly is influenced by the genetic variants. Thus, the integration of genetic variants with transcriptome data through ASE analysis enables us to detect cis-regulatory variants that may underlie phenotypic differences in a population. In other words, ASE analysis provides an opportunity to identify genes whose expressions are affected by genetic variants at the same time it gives information for mapping these cis-regulatory variants. Furthermore, ASE analysis reduces the effect of prevalent technical bias compared to DEG analysis, as ASE analysis calculates the relative abundance of alleles at heterozygous loci per individual, while in DEG analysis [10] the difference in expression of a gene is an across-samples comparison.

In addition to the classical genetic mechanisms, ASE also provides a regulatory approach to modulate gene regulation patterns in mammals [11, 12] and contributes significantly to phenotypic diversity within and between species [13, 14]. These findings indicate that genomic variants have a greater impact on transcriptome variance than previously thought [15]. Hence, it can be noted that the identified causal SNPs by ASE analysis, that regulate gene expression, may help to narrow the gap between phenotype and genotype. For instance, in a recent study, several genes with ASE were detected across adipose and muscle tissues of F1 hybrids of thin- and fat-tailed sheep breeds. Of these, two important genes (*PDGFD* and *IRF2BP2*) were reported to be related to different phenotypes between the investigated sheep breeds [16]. Cao et al. compared the ASE of genes between two diverse goat breeds and 524 genes were identified, being involved in bone development, muscle cell differentiation and lipid metabolic processes. They reported these genes as potential candidates associated with the phenotypic differences between breeds [17]. It has been demonstrated that ASE genes are involved in adipogenesis and lipid metabolism in pigs [18]. Moreover, transcriptome datasets were applied to investigate ASE in different tissues in a wide range of species, including cattle [6], pig [19], mouse [20] and chicken [21]. However, in spite of these studies, the functional role of ASE mechanism is not yet clearly elucidated, especially in non-model animals such as sheep. Accordingly, ASE analysis facilitates a deeper comprehension of the molecular mechanisms behind complex traits, such as fat deposition in sheep. To date, no comprehensive study has been conducted to identify the SNPs with a cis-regulatory effect on the expression patterns of genes involved in fat-tail formation in sheep. Previous studies have shown that genetic variants derived from RNA-Seq data are reliable and can be used for downstream analysis [4, 22] such as ASE analysis [23]. Here, by taking advantage of RNA-Seq datasets, a comprehensive ASE analysis was performed, assuming that this approach can highlight important genes associated with fat-tail development in sheep. This study attempted to provide a detailed portrait of the ASE profile that may contribute to fat-tail formation in sheep breeds. To this end, seven RNA-Seq-based studies that have compared the different fat- and thin-tailed sheep breeds, were analyzed with the same pipeline. The identified candidate genes were selected by considering the genes that showed significant differential ASE in at least two datasets. Our findings shed new light on the underlying genetic mechanisms that might influence the shape and size of fat-tail tissue in different sheep breeds.

## Material and methods

### RNA-Seq datasets

**Fig 1:**
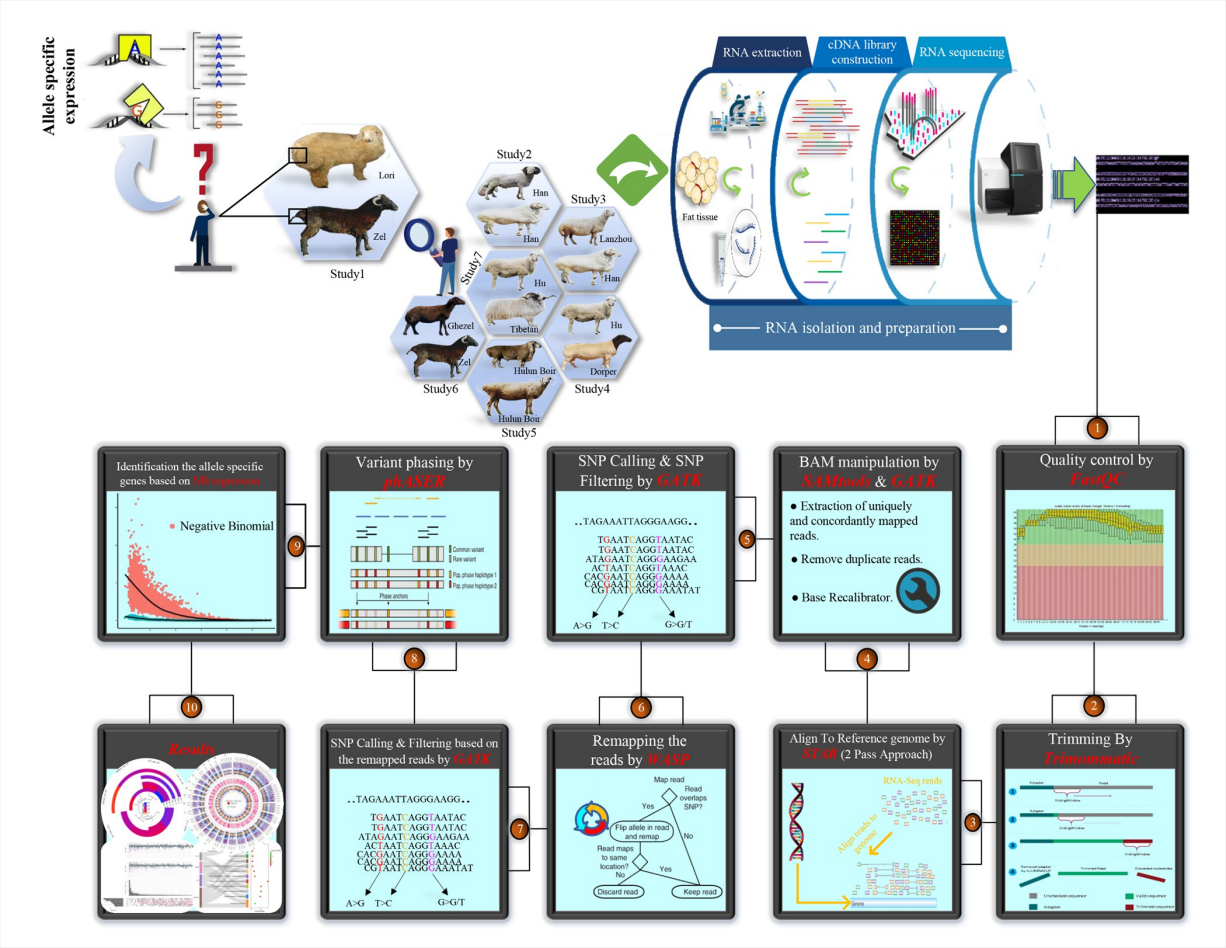
A schematic representation of the used bioinformatics pipeline to perform ASE analysis using RNA-Seq data. Datasets of the seven studies were obtained from GEO database.

In a recent study, we performed a meta-analysis on six independent RNA-Seq datasets that compared tail fat tissue of fat- and thin-tailed sheep breeds [3]. These six datasets along with one more related RNA-Seq dataset were applied in the present study to perform ASE analysis. All samples were derived from the tail fat tissue of adult male sheep with a minimum of three and a maximum of four biological replicates per breed. All studies employed transcriptomic strategies using various platforms, with most read lengths being 150 bp paired-end. All the samples were retrieved from the GEO database (Table 1) and were analyzed based on the same bioinformatics pipeline (Fig 1).

**Table 1:** Characteristics of the used studies that compared fat- and thin-tailed sheep breeds.

Study	Fat-tailed	Thin-tailed	length of reads	Average Nu of reads (million)	Platform	Accession number	Reference
Study 1	Lori (4)	Zel (4)	150	21.84	Illumina HiSeq 2000	PRJNA508203	[24]
Study 2	Han (3)	Han (3)	75	17.67	Illumina NextSeq500	PRJNA699984	[25]
Study 3	Lanzhu (3)	Han (3)	150	42.73	Illumina HiSeq 2000	PRJNA432669	[26]
Study 4	Hu (3)	Dorper (3)	150	22.41	MGISEQ-2000	PRJNA745517	[27]
Study 5	Hulun Boir (3)	Hulun Boir (3)	100	29.16	IlluminaTruSeq	PRJNA517348	[28]
Study 6	Ghezel (3)	Zel (4)	150	22.72	Illumina HiSeq2000	PRJNA598581	[29]
Study 7	Hu (3)	Tibetan (3)	150	21.81	BGISEQ-500	PRJNA792697	[30]

* Numbers in parentheses indicate biological replicates. All the samples were sequences as paired-end reads.

### Quality control and trimming

Quality control of the raw RNA-Seq reads accessed using FastQC (v0.11.9) [31]. Then, Trimmomatic (v0.35) [32] was applied to eliminate adaptor contamination and remove low-quality bases/reads using the options: TRAILING:20, MAXINFO:120:0.9, MINLEN:120. After filtering, clean reads were re-assessed by FastQC to ensure the reliability of trimming steps.

### Mapping and SNP calling

To align the clean reads against Ensembl *Ovis aries* reference genome assembly (rambouillet_v1.0.104), STAR software (v2.7.9a) was used in two-pass mode [33] with following parameters: -outFilterType BySJout, -outFilterMismatchNoverLmax 0.04, -outFilterMultimapNmax 10, -outFilterMismatchNmax 999, -outSAMmapqUnique 60 and -outFilterScoreMin 0.66. The two-pass mode was used to increase the accuracy and robustness of the alignment by improving the novel splice junction discovery. In this regard, in the first pass, STAR discovers novel splice junctions with high stringency across all the samples and inserts them into the junction database. In the second pass, it uses the novel splice sites along with known junctions to align the reads against the genome [34]. The known splice site information was obtained from the ENSEMBL GTF file (rambouillet_v1.0.104, release 106). Only concordantly and uniquely aligned reads were kept for variant calling according to the workflows and parameters recommended by GATK (v4.2.6.1) best-practice guidelines, explained as follow:

Potential PCR duplicates were removed using “MarkDuplicates” module of GATK software. Since, STAR is an RNA aligner, the alignments spanning intronic regions need to be reformatted before variant calling. To do this, SplitNCigarReads module of GATK was applied to hard clip the reads that extend into these regions. Hereafter, base recalibration to known variants based on Ensembl ovine SNP database (*ovis aries*, rambouillet) was done using BaseRecalibrator module, followed by ApplyBQSR module. Finally, variant calling was performed using GATK’s HaplotypeCaller module with a stand_call_conf and stand_emit_conf value of 30 and mbq of 25. Only biallelic SNPs were kept for ASE analysis and were filtered based on a number of standard quality metrics (Total depth of coverage<10, HomopolymerRun>5, RMSMappingQuality<40, QualitybyDepth<2, MappingQualityRankSum<-12.5 and ReadPosRankSum<-8). Spurious SNPs may result from aligning the reads to the problematic genomic regions [4]. Hence, further filtering processes were employed as follows, in an attempt to guarantee the reliability and quality of the identified SNPs for ASE analysis. Only the known variants (based on dbSNP database) with at least three supporting reads for the SNP were retained. These SNPs should not be located in problematic genomic regions including splice signal regions (within 5 bp intronic flanking), low-complexity regions (simple sequence repeats regions, ± 3 bases) and regions with bidirectional transcription.

### Remapping by WASP

RNA-Seq read depths at heterozygous loci are commonly used to measure the magnitude of the allelic imbalance in ASE analysis. This approach is prone to technical artifacts resulting from mapping biases, as reads containing non-reference alleles at heterozygous loci may not map as efficiently as reads containing reference alleles at the same site [35]. WASP tool is one of the powerful and commonly used pipelines to efficiently correct the ambiguous mapping bias for accurate ASE analysis [36]. To this end, the aligned reads were remapped following the WASP (v0.3.4) pipeline. First, uniquely mapped reads overlapping heterozygous SNPs were detected (based on the identified variants in the previous step) and the allele that is present in the read was changed to match the SNP’s other allele; if a read contains a reference allele, it is switched to the alternative allele, and if a read contains an alternative allele, it is switched to the reference allele. Then, these artificial reads were remapped and were considered as biased if they did not remap to the same genomic location efficiently. All analysis was performed based on using codes provided in https://github.com/bmvdgeijn/WASP. Finally, variant calling was performed again based on our pipeline (as described above), for the concordantly and uniquely mapped reads. The identified known SNPs from this step were selected for the ASE analysis.

### Haplotype phasing

It is strongly recommended to quantify read counts across genes based on phased variants, to enhance the accuracy and power of detecting ASE. Since a gene-based ASE can be performed by integrating effects across SNPs in the same gene, simply adding up allele counts may lead to double counting of reads and resulting in both false positives and false negatives. To overcome this limitation in the present study, a haplotype-based ASE analysis was conducted using phASER tool (v0.9.9.4). This software performs RNA-Seq-based read-backed phasing and aggregates counts across multiple SNPs (including single SNPs and haplotype blocks) to generate haplotype-level allelic read counts [37]. Hence, only uniquely mapped reads that were overlapped with heterozygous SNPs in the expressed regions of genes, were considered. Subsequently, these SNPs were phased with each other and their counts per locus were aggregated according to their respective haplotypes to estimate haplotype-level expression for each allele. To do this end, phaser.py of phASER used to count different haplotype reads. Then, phaser_gene_ae.py of this package was applied (with the flag—baseq 10) to quantify the sequencing reads overlapping heterozygous sites within protein coding genes based on gene bed file of Ensembl version 104 and gene level haplotypic expression matrix generated.

### Differential ASE

To identify significant differences in allelic imbalances between the fat-tail of the sheep breeds, a gene-based ASE analysis was performed considering the null hypothesis that ASE values for a specific gene should be roughly equivalent between two groups. To this end, all samples of each breed of the seven studies were combined and two groups including fat- and thin-tailed compared. Taking into account the count-based nature of the data, a logistic regression model using a negative binomial distribution was adapted to evaluate whether the gene had allele-specific expression as well as its allelic imbalance differed between fat- and thin-tailed sheep breeds. It is worth noting that unlike the Poisson distribution, which assumes equal mean and variance, the negative binomial distribution can be considered a generalization of a Poisson distribution to allow for overdispersion, making it a more appropriate model for count-based dataset. In fact, negative binomial distribution introduces an extra parameter to model the overdispersion. This parameter captures the variability not explained by the Poisson distribution. In cases where there is no overdispersion, the negative binomial distribution simplifies to the Poisson distribution [36]. If the read counts for the reference and alternative alleles be denoted by ref and alt, respectively, the following model was used: glm.nb(ref ~ group, offset = (log(ref + alt))). In this model, offset adjust unequal library sizes across different samples. Also, group indicate the fat- and thin-tailed sheep breeds. Multiple testing correction was performed with the false discovery rate (FDR) method, and significant ASE genes were defined as those with adjusted p-values < = 0.1. For the last filtering step, a significant ASE gene should be observed in the samples of at least two studies to be considered a functional ASE gene. All the statistical analysis and visualization of the results were performed using R software (v4.3.2).

### Functional analysis

To gain insights into the potential function of the differential ASE genes, functional enrichment analysis was performed using the EnrichR tool [38]. Biological processes and KEGG pathways with an FDR < 0.05 were considered significant. The significance level was determined using the gene ratio and adjusted p-value. The term "gene ratio" refers to the proportion of genes of interest present in a particular gene set or pathway relative to the total number of genes in that set. The adjusted p-value accounts for multiple hypothesis testing, helping to control the increased chance of false positive results when performing multiple statistical tests, thus providing a more accurate measure of statistical significance. The biological processes as well as KEGG pathways with FDR<0.05 were considered as significant terms.

## Results

### RNA-Seq data analysis

In total, more than 1,137 million reads, with an average of 25 million reads per sample, were analyzed. Of these, ~557 and ~580 million reads were related to fat- and thin-tailed breeds, respectively. The read counts ranged from eight to 50 million reads. After trimming and cleaning the RNA-Seq datasets, ~1,132 million reads were retained, of which ~576 and ~556 reads belonged to thin- and fat-tailed breeds, respectively. Only ∼0.004% of the reads were removed after filtering, reflecting the high quality of the data. Alignment rate of the clean reads against the reference genome ranged from 77 to 99% with an average rate of 93%. Of the aligned reads, ~974 million reads (~86%) were uniquely mapped to the genome. Details of the number of raw and clean reads as well as mapping statistics of different samples are provided in S1 File.

### SNP calling

**Fig 2:**
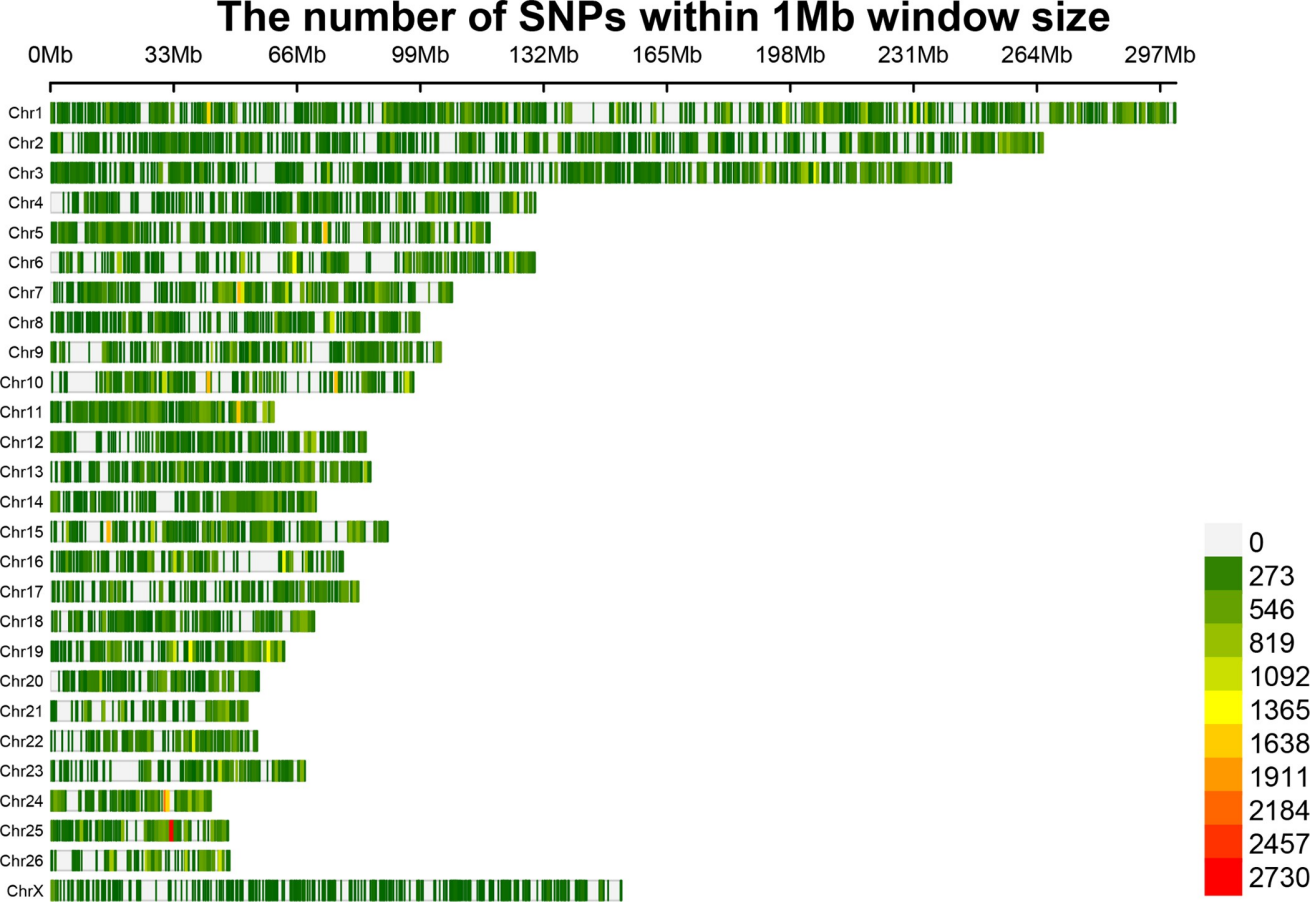
The distribution of all identified heterozygous SNPs across the chromosomes of the reference sheep genome.

According to our RNA-Seq-based SNP discovery pipeline, a total of 1,415,762 known SNPs were identified in all samples after filtering. Then, heterozygous loci were extracted and subjected to remapping the reads by WASP approach to improve the accuracy of SNP calling. SNP discovery was performed again and 442,229 known heterozygous SNPs were discovered across all samples, which were applied for differential ASE analysis. Of these, 322,468 (72.8%) and 120,042 (27.2%) SNPs were transitions and transversions, respectively. The average number of SNPs per sample was 33,822. Of these, 270,437 SNPs were detected in the fat-tailed breeds and 341,523 SNPs in the thin-tailed group. The number of SNPs per chromosome varied from 50,055 (Chr1) to 5,512 (ChrX) with an average of 16,379 per chromosome (Fig 2).

### ASE analysis

**Fig 3:**
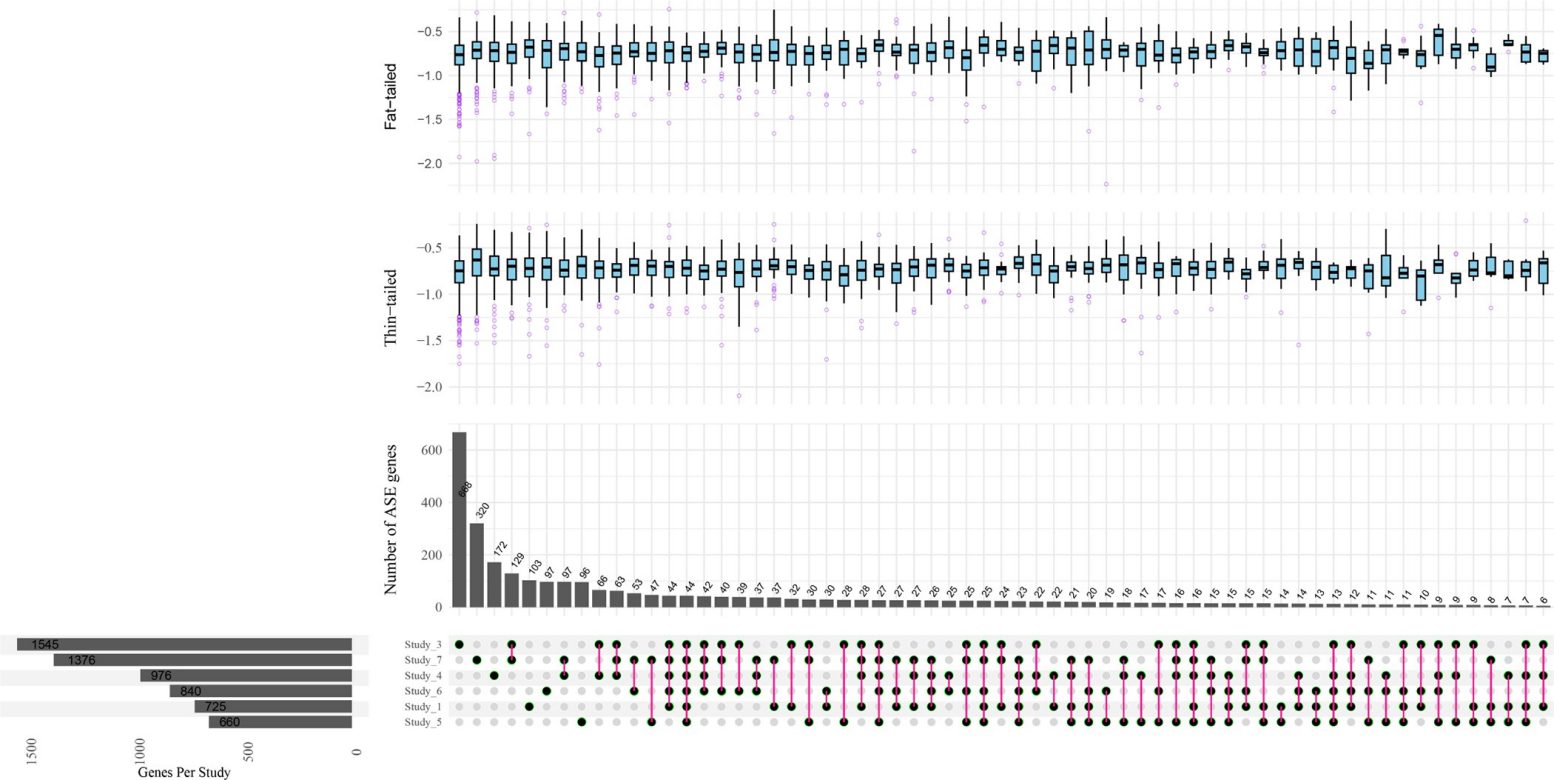
UpSet plot of the common genes that were subjected to ASE analysis among the different studies. The bar plots indicate the number of genes shared by specific studies. The horizontal bar chart at the bottom left represents the total number of genes found in each study. The box plots indicate the distribution of allelic ratio values for each breed. Study_2 is excluded as it did not have enough genes to be displayed.

**Fig 4:**
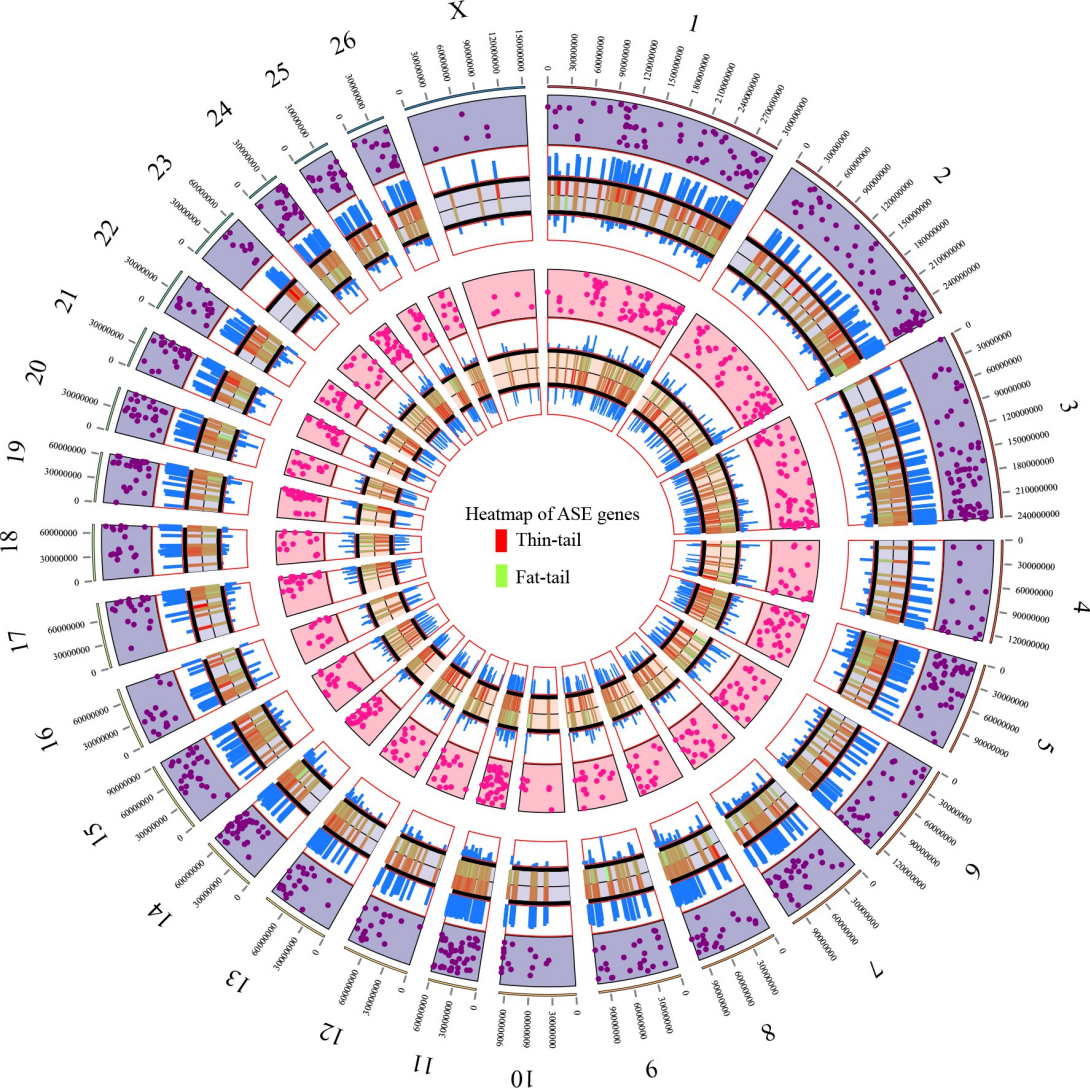
Circos plot displaying the genome-wide distribution of the ASE analysis results. The outermost ring shows chromosome numbers. The analyzed genes were divided into two groups based on their allelic ratio: the genes with an average allelic ratio above 0.5 in the thin-tail breeds are shown in the first inner ring (purple points). While the genes with an average allelic ratio above 0.5 in fat-tail sheep breeds are presented in the second inner ring (pink points). The heatmaps are accompanied by histograms that show the average allelic ratio of each gene in both tail fat groups. The green and red colors of heatmap indicate the higher allelic ratio in thin- and fat-tailed sheep breeds, respectively.

**Fig 5:**
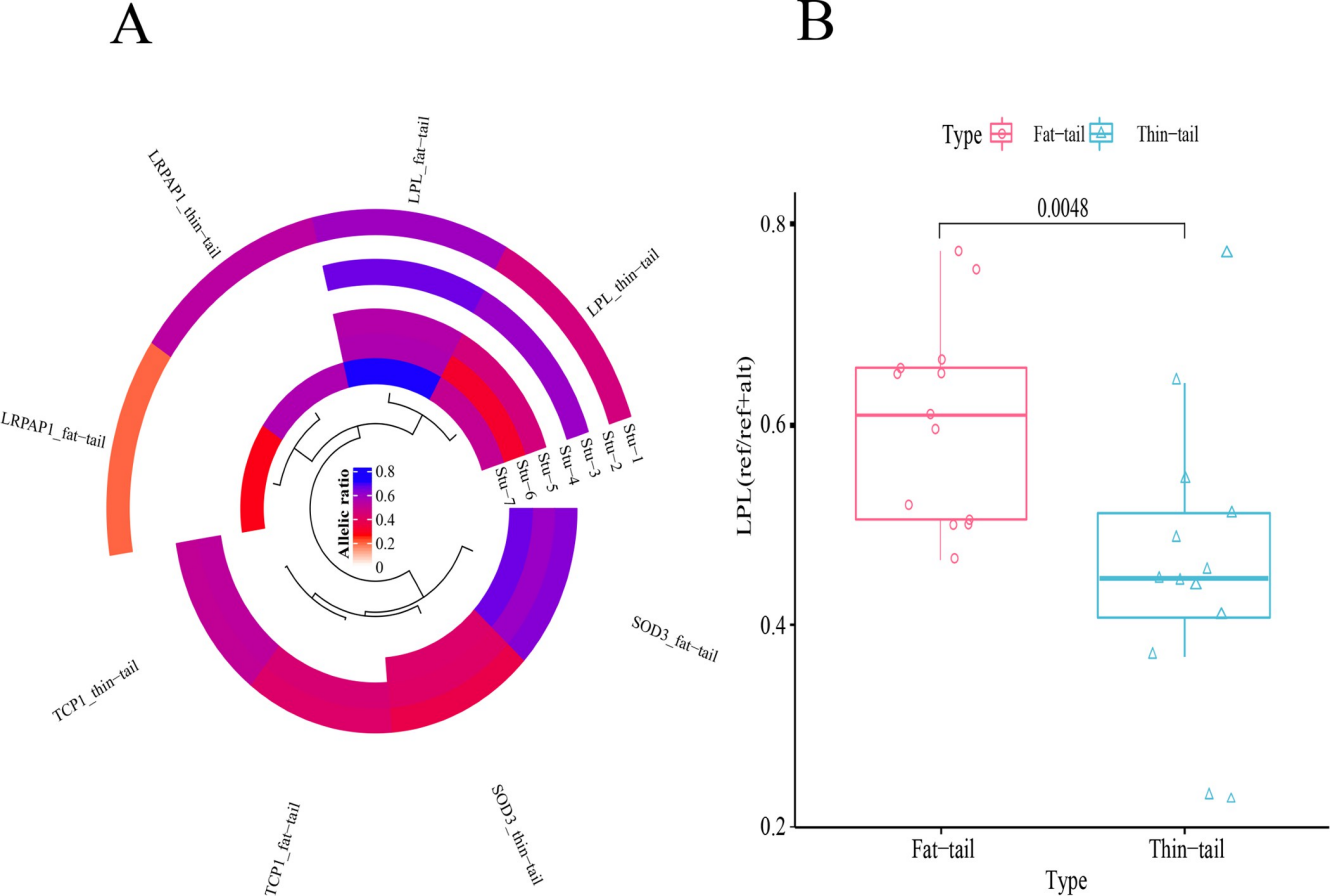
Allelic ratio of the significant differentially ASE genes in fat- and thin-tailed sheep breeds. A) Heatmap representation of the allelic ratio for the four significant ASE genes in the used studies. The color scale indicates the intensity of higher expression of the reference allele. B) Box plot related to average allelic ratio of *LPL* gene in the fat-tail and thin-tailed breeds (FDR = 0.0048).

After aggregating the SNPs in haplotypes within the genic regions using PhASER [37], 2,995 genes were identified and subjected to differential ASE analysis. Fig 3 shows an UpSet plot of the intersection of these genes among the different studies. Statistical analysis revealed 115 differential ASE genes with a p-value <0.05 that were observed in at least one study. To improve the reliability of results, a set of stringent filtering criteria (Material and Methods) was applied and four genes (*TCP1*, *LPL*, *LRPAP1* and *SOD3*) were selected based on the ASE significance threshold of FDR< = 0.1, also requiring that these ASE genes were identified in at least two studies. Of these, *LPL* was identified presenting ASE in five studies, *SOD3* in three and the other two ASE genes were identified in two studies (S2 and S3 Files). To be sure that these genes are not imprinted genes in sheep, Geneimprint database (http://www.geneimprint.com/site/home) was investigated and it was found that none of them were imprinted genes. Genome-wide distribution of ASE results along with allelic ratio of the genes in both breeds is shown in Fig 4 as a Circos plot. Furthermore, Fig 5 shows the allelic ratio of the significant ASE genes across the different studies. As it is evident from Fig 5A, allelic ratio of the genes has the similar pattern in different studies in fat- and thin-tailed sheep breeds, which emphasized the reliability of the results.

### Functional enrichment analysis

**Fig 6:**
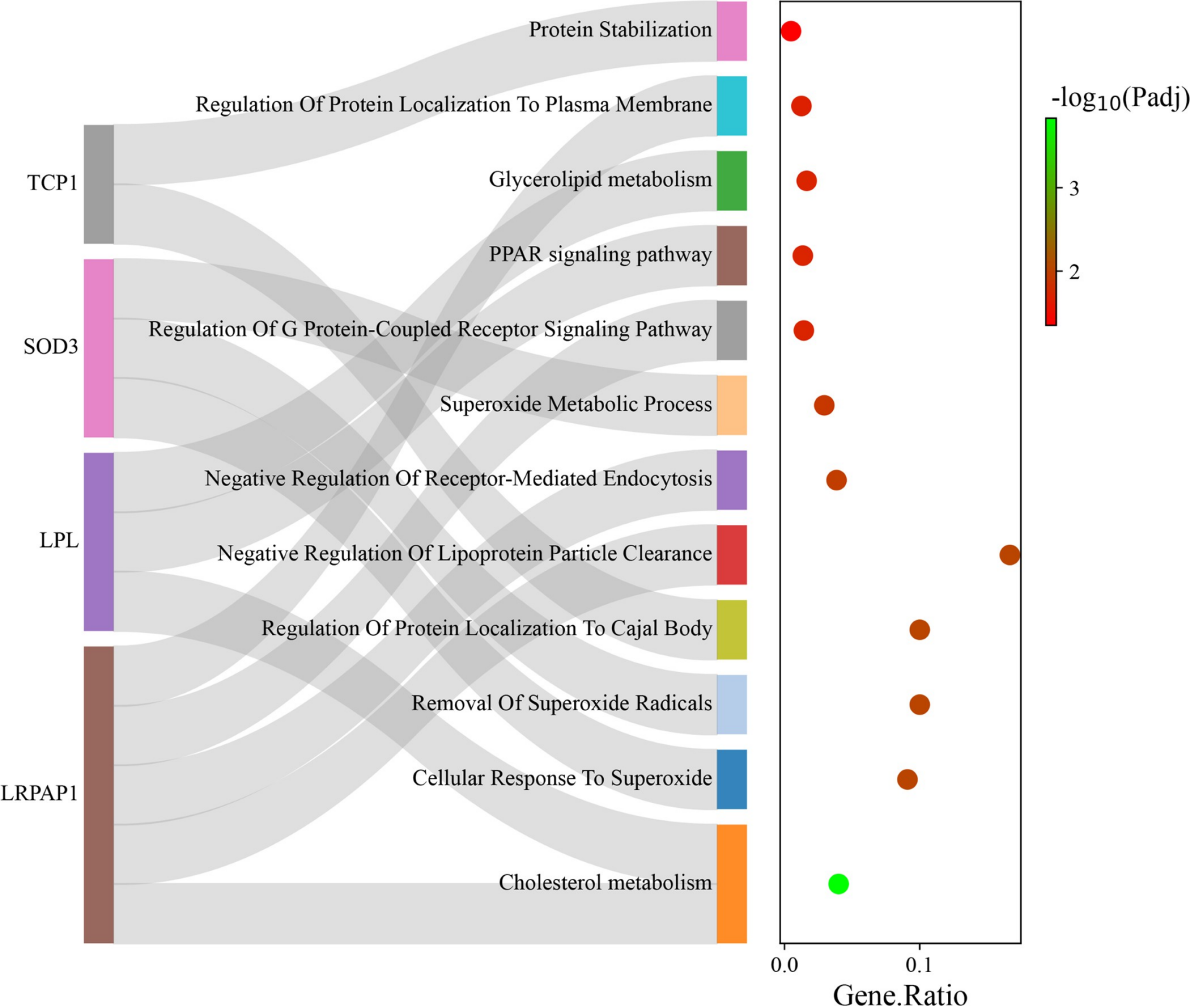
Sankey plot related to the results of functional enrichment analysis. The plot shows the connections between the selected significant genes (left) and the enriched biological terms (right).

To explore the potential biological function of the genes with significant ASE, functional enrichment analysis was performed. In total, 44 biological processes and three KEGG pathways were significantly enriched with FDR < 0.05. It is noteworthy that several terms were directly related to fatty acid metabolism such as cholesterol metabolism (*LPL* and *LRPAP1*), PPAR signaling pathway (*LPL*) and negative regulation of lipoprotein particle clearance (*LRPAP1*) (Fig 6). S4 File provides the complete list of the functional enrichment analysis results.

## Discussion

ASE is one of many genetic mechanisms that contributes to the development of complex traits and phenotypic variation among organisms [39, 40]. Hence, it is well documented that allelic imbalance of genes can be the result of selection, with the functional consequence for adaptive phenotypic plasticity between breeds [41]. With the advantage of traditional bulk transcriptome analysis in mind, ASE analysis enable us to quantify the relative expression of alleles of genes. Therefore, we can identify the genes with similar total expression patterns between two groups of samples, while the expression of their alleles is not similar. Actually, ASE analysis is a useful approach to infer cis-regulatory variants associated with this expression pattern and enables us to decipher the genotype to phenotype map underlying complex traits. However, the exploration of genes containing ASE in the context of fat-tail formation in sheep remains relatively limited in existing literature. Previous studies have predominantly focused on classical transcriptome [24–30, 42] or genomic data analysis [4, 43–46]. Therefore, there is a gap between genetic variants and the molecular genetics behind the fat-tail development in sheep, and ASE analysis may provide novel insights to help close that gap. In this regard, to better understand the genetic mechanisms related to the phenotypic differences between fat- and thin-tailed sheep breeds, seven independent RNA-Seq datasets (fat-tail tissue) were analyzed to detect differential ASE genes at the whole-genome level. This study was performed to propel future research into important cis-acting genetic SNPs that may shape the fat-tail tissue of sheep. According to our previous studies [4, 47–49], an RNA-Seq based genetic variant discovery approach was applied to identify unbiased SNPs. Generally, more genetic variants (~ 21%) were detected in thin- than fat-tailed sheep breeds. This finding is in agreement with the hypothesis that wild ancestors of domestic sheep had thin tails and that fat-tailed breeds evolved as an environmental adaptation response after domestication [24, 50–52]. Therefore, it is expected that thin-tailed breeds, being the ancestral population, have greater genetic diversity. However, it is worth noting that the more SNPs in thin-tailed sheep breeds can be attributed to the faster evolution than fat-tailed breeds, which need to be further investigated.

On the other hand, to accurately estimate the allelic expression of genes, various aspects have to be considered during ASE analysis. Here, a reliable approach, the WASP method, was applied to remove the mapping bias favoring reference alleles [36], an important aspect to be controlled when investigating ASE [10]. It is reported that mapping bias correction using the WASP approach leads to obtaining extremely low false positive results in ASE analysis [53, 54]. Another aspect to take into account is the sparse and over-dispersed nature of count data [55], which is very common in allelic expression data. Assuming a Poisson or standard binomial distribution to test differential ASE genes can cause false positive results [56]. To avoid this, we applied a negative binomial distribution that can handle the variance independent of the mean using an additional parameter to explain the overdispersion in the data [57]. However, the key to our approach is using seven independent RNA-Seq datasets that provide an ideal opportunity to identify the common ASE genes associated with fat-tail formation in different sheep breeds. In other words, observing an ASE gene in some relevant studies makes it more likely to be involved in fat-tail development. Altogether this approach led to the identification of 115 genes with evidence of differential ASE (p-value <0.05). After adjustment for multiple comparisons and considering the ASE genes that were presented in at least two studies, four genes (*TCP1*, *LPL*, *LRPAP1* and *SOD3*) were identified, which are predominantly involved in lipid metabolism processes such as "cholesterol metabolism”, “PPAR signaling pathway” and “negative regulation of lipoprotein particle clearance”. All these terms are directly related to fat deposition. For instance, it is well known that TFs of the PPAR play important roles in the clearance of cellular lipids via the regulation of many genes involved in fatty acid oxidation [24]. Lipoprotein particles, such as LDL (low-density lipoprotein) and VLDL (very-low-density lipoprotein), transport cholesterol and triglycerides in the bloodstream. Negative regulation of lipoprotein particle clearance refers to processes that decrease the removal of these particles from the blood. This can lead to higher levels of circulating lipoproteins, contributing to fat deposition [58].

Of the identified differential ASE genes, *LPL* stands out for the following reasons: First, it was observed as ASE in five different studies, which reinforces its potential regulatory role as ASE gene in tail fat deposition of sheep. It is essential to emphasize that the pattern of the allelic ratio of this gene was the same in the samples of each phenotypic group (Fig 5). Second, *LPL* is reported as ASE in several previous studies, showing consistency with our findings. Wang et al. used male Texel and female Kazakh to generate a hybrid population (n = 15). These F1 hybrids were applied to investigate the ASE genes in adipose tissue between thin- and fat-tailed breeds within the population. Fifteen ASE SNPs were identified in *LPL*, reported as an important ASE gene associated with lipid metabolism [16]. Moreover, ASE of *LPL* has been demonstrated in bovine muscle [6] and human tissues [59]. In this regard, *LPL* was reported as a gene containing breed-specific SNPs in comparison of two Iranian sheep breeds with different fat-tail phenotype (Lori vs Zel) [4]. Last but not least, *LPL* is closely related to lipid metabolism which makes it a promising candidate involved in fat-tail development through ASE mechanism. For example, *LPL* is reported as the most important factor affecting chicken fat deposition [60]. Moreover, the higher *LPL* activity was related to the faster abdominal adipose tissue fat deposition of broilers than laying hens [61]. *LPL* is a rate-limiting enzyme involved in the hydrolysis of plasma triglyceride (TG) (from TG-rich lipoproteins), thereby generating free fatty acids and glycerol [62]. These free fatty acids can be used as ligands and activators of peroxisomal proliferator activated receptor (PPAR) family of nuclear hormone receptors, which plays a key role in the metabolism of fatty acids and lipoproteins. Three PPAR isoforms have been identified including PPAR-α, PPAR-δ and PPAR-γ. Of these, α and δ stimulate fatty acid oxidation and γ activates lipid storage and adipogenesis [63, 64]. It is reported that products of *LPL* activity are an important source of PPAR-α and -δ ligands [65]. In this context, a possible hypothesis can be that genetic divergence and possibly adaptation to different environments induced genetic variants causing the difference in expression of *LPL* alleles. Different alleles of *LPL* may result in proteins with various catalytic efficiencies that lead to phenotypic variation of sheep fat-tail. This process may operate in concert with other molecular mechanisms to shape the different fat-tail phenotypes in sheep. A point worth emphasizing is that bulk transcriptome analysis only considers the total expression of genes and genes with ASE cannot be detected, while their total expression is similar in both conditions of interest. *LPL* can be considered as a typical example, as we previously showed that there is no significant difference for *LPL* expression level in the fat-tail of sheep breeds with different fat deposition contents using real-time PCR [62]. In this regard, *LPL* was not reported as DEGs between fat- and thin-tailed sheep breeds in most of the previous relevant studies [24, 26, 27, 29, 30] as well as in our previous meta-analysis of RNA-Seq datasets [3]. In line with this trend, *LPL* was not differentially expressed in subcutaneous and omental fat depots, between two cattle breeds with different fat depositions [66]. In the present study, a significant allelic imbalance of *LPL* was observed in most of the investigated datasets (>75% of samples). Therefore, in spite of the same expression pattern of *LPL* between fat- and thin-tailed sheep breeds, differences between the expression of alleles of this gene may contribute to the phenotypic heterogeneity of fat-tail among sheep breeds. This finding indicates that different molecular mechanisms have to be considered to identify the effective factors associated with a multi-layered biological phenomenon, such as fat-tail development.

The other important differential ASE gene (*SOD3*) is a member of a key family of enzymatic antioxidants, named superoxide dismutase family (including three isozymes *SOD1*, *SOD2* and *SOD3*). *SOD3* catalyzes the dismutation of two superoxide radicals into hydrogen peroxide and oxygen, which are less harmful to cells [67]. High-level expression of *SOD3* in adipose tissue and adipocytes has been demonstrated in mice [68]. In this regard, it is well known that *SOD3* is associated with fat deposition in animals [69, 70] and humans [71]. Furthermore, the role of *SOD* family, especially *SOD3*, in lipid metabolism and obesity has been previously explained in mice [72], chicken [73, 74], beef [75], dairy cattle [76], and pig [77]. For example, *SOD* knockout mice were more obese with larger adipose tissue and more TG than wild mice [68]. Here, unequal expression of *SOD3* alleles was significantly detected in three studies which highlight its importance in fat deposition in the tail of sheep. It can be interesting to notice that a previous study revealed that *SOD3* silencing increased the expression of genes involved in the PPAR pathway and stimulated the accumulation of TG in human adipocytes [68]. As discussed above, *LPL* provides the ligands of PPAR (free fatty acids), which tempts us to suggest there as a possible interaction between these genes (*LPL* and *SOD3*) to promote tissue fat deposition and affect fat-tail phenotype. Although this finding is not validated at the experimental level, it can motivate further studies to investigate the proposed mechanism.

Two other differential ASE genes (*LRPAP1* and *TCP1*) were observed only in two datasets, however, their roles in lipid metabolism have been described in the previous studies. Interestingly, both genes are reported as genes showing ASE in ovine brown adipose tissues [78]. LDL receptor-related protein associated protein-1, *LRPAP1*, encodes a receptor-associated protein (RAP) that regulates circulating cholesterol in mice [79]. This gene binds to both *LRP1* and *LRP2* on the cell surface in the presence of calcium ions, which are the receptors that mediate the uptake of lipoproteins and various lipids [80]. In a recent study, a copy number variation screening was performed in fat- and thin-tailed sheep breeds. Association analysis detected a significant region associated with fat metabolism harboring *LRPAP1* gene [81]. Also, the identified genetic variants in *LRPAP1* are reported to be closely associated with high plasma lipid levels [82]. T-complex protein 1, *TCP1*, is a subunit of *TCP1*containing ring complex (TRiC) [83], which is a molecular chaperone that assists in the folding of newly synthesized proteins. Also, it plays a role in cell growth, proliferation and apoptosis in eukaryotes [84]. Important role of *TCP1* in the remodeling of cytoskeletal protein (like actin and tubulin) has been documented [85]. On the other hand, it is well known that remodeling of cytoskeletal protein is one of the most important biological processes that occurred during the adipogenesis. Hence, potential role of this gene in lipid metabolism and fat deposition can be explained, as it is reported in bovine intramuscular fat accumulation [86]. Furthermore, Taga et al. investigated the key cellular and molecular events in the growth of adult adipose tissue in bovine fetal at different days post-conception (dpc). They identified proteins such as *TCP1* at 110 and 180 dpc, which may regulate the proliferation of adipocyte precursors by controlling cell cycle progression and/or apoptosis or delaying PPARγ-induced differentiation [87]. It has also been reported that the subunits of TRiC activate the unfolded protein response (UPR), which enables a coordinated regulation of lipid metabolism and overall levels of cellular energy [88]. The results of the present study suggest that deviation from the general model of biallelic expression can provides an efficient way to understand the phenotypic variation as well as fat deposition pattern in tail of sheep breeds.

## Conclusion

This study provides the first comprehensive analysis of ASE across fat-tail of 45 samples belonging to seven independent studies. Overall, a higher genetic diversity was observed in the thin-tailed breeds compared to the fat-tailed breeds. We identified four key candidate genes, *LPL*, *SOD3*, *LRPAP1* and *TCP1*, showing significant differential ASE between fat- and thin-tailed sheep breeds. These genes were of great significance, as they were closely related to fat deposition. Among these genes, *LPL* has a well-known involvement in fat deposition, being detected as a differential ASE gene in most of the investigated studies. Moreover, *LPL* was reported as an ASE gene in previous studies, which makes it a promising candidate for fat-tail formation in sheep. Taken together, these results suggested that ASE can be considered as a potential regulatory mechanism behind the fat-tail development and the identified differential ASE genes may contribute to the shape of tails in sheep. Thus, the cis-regulatory mechanisms underlying these genes’ expression patterns can be further explored. Therefore, the proposed candidate genes, especially LPL, are worthy of further investigation to explain the phenotypic variation of sheep fat-tail and improve our current understanding of the process of fat-tail development.

## Supporting information

S1 File Details of the clean reads, uniquely mapped reads, multi mapped reads, percent of mapped reads per sample, after using our pipeline. (XLSX)

S2 File List of differential ASE genes and their allelic ratio (Reference / Alternative) across all samples. (XLSX)

S3 File The identified genotypes/haplotype of the identified four ASE genes (*TCP1*, *LPL*, *LRPAP1* and *SOD3*) in different samples. (XLSX)

S4 File List of the identified biological processes and KEGG pathways related to the genes with significant ASE. (XLSX)
